# *Clinacanthus nutans* (Burm. f.) Lindau Extract Inhibits Dengue Virus Infection and Inflammation in the Huh7 Hepatoma Cell Line

**DOI:** 10.3390/antibiotics13080705

**Published:** 2024-07-28

**Authors:** Kanyaluck Jantakee, Suthida Panwong, Pachara Sattayawat, Ratchaneewan Sumankan, Sasithorn Saengmuang, Kiattawee Choowongkomon, Aussara Panya

**Affiliations:** 1 Department of Biology, Faculty of Science, Chiang Mai University, Chiang Mai 50200, Thailand; kanyaluckjan@gmail.com (K.J.); pachara.sattayawat@cmu.ac.th (P.S.); 2 Doctoral of Philosophy Program in Applied Microbiology (International Program), Faculty of Science, Chiang Mai University, Chiang Mai 50200, Thailand; suthida_pan@cmu.ac.th; 3 Cell Engineering for Cancer Therapy Research Group, Faculty of Science, Chiang Mai University, Chiang Mai 50200, Thailand; 4 Graduate Master’s Degree Program in Biology, Faculty of Science, Chiang Mai University, Chiang Mai 50200, Thailand; ratchaneewan_sum@cmu.ac.th (R.S.); sasithorn-mind@hotmail.com (S.S.); 5 Department of Biochemistry, Faculty of Science, Kasetsart University, Bangkok 10900, Thailand; kiattawee.c@ku.th

**Keywords:** dengue virus, *Clinacanthus nutans*, antiviral agent, pro-inflammatory cytokine, anti-inflammation, NF-κB signaling

## Abstract

Dengue virus (DENV) infection has emerged as a global health problem, with no specific treatment available presently. *Clinacanthus nutans* (Burm. f.) Lindau extract has been used in traditional medicine for its anti-inflammatory and antiviral properties. We thus hypothesized *C. nutans* had a broad-ranged activity to inhibit DENV and the liver inflammation caused by DENV infection. The study showed that treatment using *C. nutans* extract during DENV infection (co-infection step) showed the highest efficiency in lowering the viral antigen concentration to 22.87 ± 6.49% at 31.25 μg/mL. In addition, the virus–host cell binding assay demonstrated that *C. nutans* treatment greatly inhibited the virus after its binding to Huh7 cells. Moreover, it could remarkably lower the expression of cytokine and chemokine genes, including *TNF-α*, *CXCL10*, *IL-6*, and *IL-8*, in addition to inflammatory mediator *COX-2* genes. Interestingly, the activation of the NF-κB signaling cascade after *C. nutans* extract treatment was dramatically decreased, which could be the underlying mechanism of its anti-inflammatory activity. The HPLC profile showed that gallic acid was the bioactive compound of *C. nutans* extract and might be responsible for the antiviral properties of *C. nutans*. Taken together, our results revealed the potential of *C. nutans* extract to inhibit DENV infection and lower inflammation in infected cells.

## 1. Introduction

Dengue virus (DENV) infection is a globally important public health problem. It is estimated that approximately 390 million DENV cases are reported per year. Among these, people in Asia account for up to 70% of the worldwide disease burden [1,2]. DENV belongs to the family of Flaviviridae, with a positive single-stranded RNA genome. It encodes for 11 proteins including capsid, pre-membrane, and envelope (structural) proteins, in addition to non-structural proteins (NS1, NS2A, NS2B, NS3, NS4A, NS4B, and NS5) [3]. There are four serotypes, DENV-1, DENV-2, DENV-3, and DENV-4, which are co-circulated in the endemic area and transmitted via mosquito vectors in *Aedes* genus. The infections demonstrate a wide range of symptoms ranging from mild disease to dengue fever and dengue hemorrhagic fever (DHF). Dengue shock syndrome (DSS) is the most severe disease, with a life-threatening manifestation in which vascular leaking can occur [4]. Nowadays, there is no specific drug for DENV treatment, while the available vaccine, Dengvazia, cannot fully protect against all DENV serotypes. Furthermore, other developing vaccines, i.e., the Qdenga vaccine and TV005 tetravalent live-attenuated dengue vaccine, are not yet approved by the US FDA and are undergoing clinical trials [5]. Thus, the search for anti-DENV agents is still needed to lower its severity and mortality, especially in endemic countries.

DENV can infect diverse types of immune cells including macrophages, lymphocytes, dendritic cells, and organs such as the lungs, liver, and brain, which is associated with the complication of severe symptoms [6,7,8]. The infection can promote inflammation and antiviral responses via the production of cytokines and chemokines to eliminate viral particles. The uncontrolled response via massive cytokine/chemokine production or a cytokine storm occurs in addition to the acute inflammation that participates in the pathogenesis of DENV infection. Hepatocytes are one of the critical targets of DENV. The inflammation of liver tissue is observed in severe cases of DENV and is correlated with an accumulation of pro- and anti-inflammatory cytokines such as tumor-necrosis factor alpha (TNF-α), interferon-gamma (IFN-γ), interleukin-10 (IL-10), transforming growth factor-beta (TGF-β), and regulated upon activation, normal T-cell expressed and secreted (RANTES), produced by mononuclear cell types [9,10]. Concordantly, DENV infection in HepG2 cells showed an increase in the innate immune response via toll-like receptor 3 (TLR3), toll-like receptor 8 (TLR8), retinoic acid-inducible gene I (RIG-I), and melanoma differentiation-associated gene 5 (MDA5) and upregulated pro-inflammatory gene expression, i.e., *IL-6*, *IL-8*, and *RANTES* [11]. Moreover, in Brazil, the increases in IL-10, macrophage migration inhibitory (MIF), and C–X–C-motif chemokine 10/interferon gamma-induced protein 10 (CXCL10/IP-10) during the acute phase in severe cases have been reported to be correlated with hepatomegaly and liver dysfunctions [12]. The liver is considered a site of DENV tissue tropism. Several receptor types, such as the scavenger receptor B type 1 (SRB1), erb-b2 receptor tyrosine kinase 2 (ERBB2), and insulin-like growth factor 1 receptor (IGF1R), have been reported to respond to DENV infection in liver cells, promoting pathogenic mechanisms [13,14]. Although dengue is an infectious disease, its severity is often due to an overreaction of the immune response, resulting in a cytokine storm, which can be fatal. An antiviral agent that solely targets the virus without reducing inflammation might not be sufficient to mitigate the risk of severe disease. Taken together, a promising treatment for DENV infection must not only control DENV replication but also reduce serious symptoms such as massive cytokine production, liver inflammation, and dysfunction during the critical phase.

Bioactive compounds from natural products are used in traditional healthcare worldwide. Unlike small synthetic compounds or drugs, the herb/plant extract provides broad-spectrum pharmacological properties since it contains numerous compounds and secondary metabolites which can target different proteins through different pathways. Accordingly, herb/plant extracts provide a high possibility for dual antiviral and anti-inflammatory activities, which is promising for DENV treatment. *Clinacanthus nutans* (*C. nutans*), a member of Thailand’s national list of essential medicines [15], has been reported for its pharmacological activities, i.e., antiviral, anti-inflammatory, antioxidant, anti-cancer, anti-diarrheal, anti-diabetic, and renoprotective activities. It has been used to treat herpes simplex virus-induced lesions as well as inflammatory conditions, i.e., rheumatism, sprains, injuries, contusion, and hematoma [16,17]. Its antiviral properties are also reported to inhibit infection by human papillomavirus and varicella zoster [16,18]. Recently, the antiviral effect of *C. nutans* extracts has been reported to inhibit chikungunya virus by inhibiting viral progeny release [19] and SARS-CoV-2 by binding to viral protease and angiotensin-converting enzyme 2 (ACE2) receptor proteins [20]. However, no study has reported on the antiviral and anti-inflammation activity of *C. nutans* against DENV, particularly in hepatocyte cells. In this study, the antiviral effect of *C. nutans* extract on inhibiting DENV serotype 2 (DENV-2) was demonstrated in the Huh7 hepatocellular carcinoma cell line. The anti-inflammation was evaluated according to the reduction in inflammatory cytokines, and the suppression of inflammation via the inhibition of the nuclear factor kappa B (NF-κB) signaling pathway was also studied. This study is the first to demonstrate the potential of *Clinacanthus nutans* extract and explore the mechanisms by which the extract affects DENV during the early stages of its life cycle, providing crucial information for treatment regimens.

## 2. Results

### 2.1. Inhibition of DENV-2-Infected Huh7 Hepatoma Cells Upon Treatment with Clinacanthus nutans Extract

**Figure 1:**
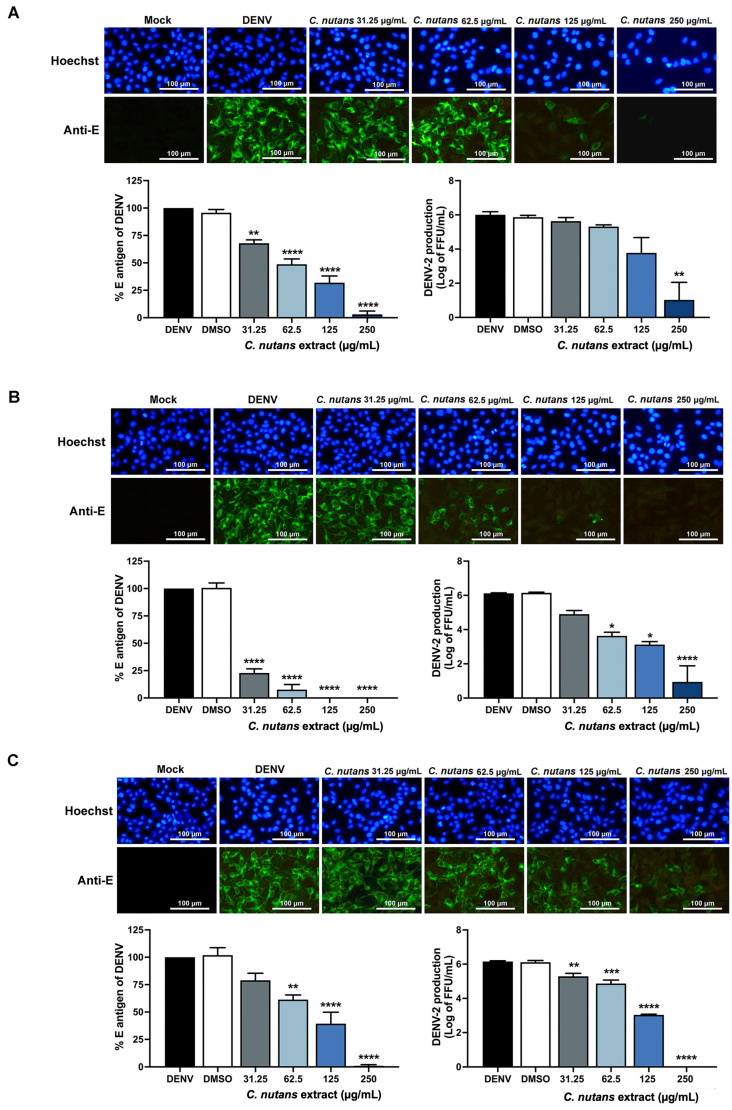
Antiviral activity of *C. nutans* extract against DENV-2 in Huh7 hepatoma cells. The *C. nutans* extract (31.25–250 μg/mL) was added to the cells at different steps including before infection (**A**), co-infection (**B**), and post-infection (**C**). After 48 h of infection, the infected cells were measured by using IFA, cell-based ELISA assay, and virus titration compared to that of the non-treatment control (* *p* < 0.05; ** *p* < 0.01; *** *p* < 0.001; and **** *p* < 0.0001).

The cytotoxicity of *C. nutans* extract was evaluated in Huh7 cells to select a non-toxic dose. The result showed that *C. nutans* extract is highly safe, with a CC50 of 632.4 μg/mL (Supplementary Materials, Figure S1). The antiviral activity was then tested at a concentration ranging from 31.25 to 250 μg/mL in Huh7 cells to inhibit DENV-2 infection. The *C. nutans* extract was added at different steps of infection: before-infection, co-infection, and post-infection conditions. The result showed that *C. nutans* extract inhibited DENV infection in all three conditions in a dose-dependent manner judged by IFA, cell-based ELISA, and an FFU assay (Figure 1A–C). Among the three conditions, addition of the extract at the co-infection step showed the greatest effectiveness in inhibiting viral infection. Adding *C. nutans* extract at a concentration of 125 μg/mL during the co-infection step could fully protect Huh7 cells from infection (Figure 1B), whereas adding the extract during the before-infection condition (Figure 1A) and post-infection condition (Figure 1C) reduced the percentage of E antigen to 31.95 ± 10.62% and 39.32 ± 18.19%, respectively. At the lowest concentration (31.25 μg/mL) of *C. nutans* extract, it reduced the percentage of E antigen to 22.87 ± 6.49% at the co-infection step (Figure 1B). Concordantly, the IFA showed a dramatic reduction in infected cell number as well as the production of new viral progeny after treatment with *C. nutans* extract during the co-infection step, greater than that observed under the other conditions. These results suggest that *C. nutans* extract inhibited DENV infection effectively at the early step of viral infection.

### 2.2. Inhibition of DENV-2 Infection in the Early Steps of Infection by C. nutans Extracts

**Figure 2:**
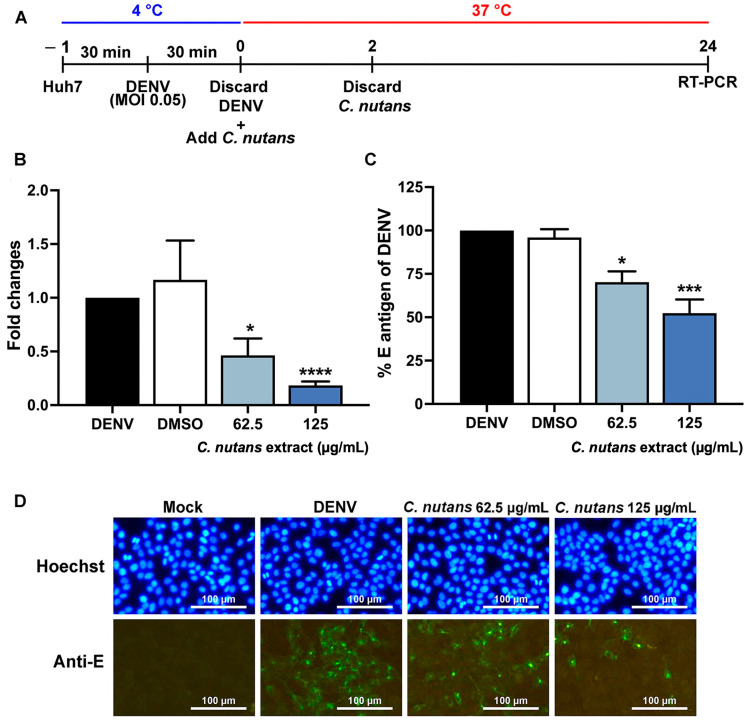
Efficacy of *C. nutans* extracts on an early step of infection. The scheme of extract treatment during the early step of DENV-2 infection (**A**). After 24 h of infection, the genomic RNA was measured using qRT-PCR comparing between the infected cells with or without *C. nutans* extract (62.5–125 μg/mL) (**B**). The reduction in DENV-2 was confirmed by using ELISA (**C**) and IFA assay after 48 h of infection (**D**). The results show the mean ± SEM values from three independent experiments (* *p* < 0.05, *** *p* < 0.001, **** *p* < 0.0001).

Since *C. nutans* extract efficiently inhibited DENV-2 infection at the co-infection step, we also hypothesized that *C. nutans* extract might affect the early step of infection. To prove this hypothesis, we tested whether the virus could affect RNA replication using qRT-PCR after a viral binding step. The virus was allowed to bind to Huh7 at 4 °C and then treated with *C. nutans* extract for 2 h during the early step of infection (Figure 2A). At 24 h of infection, DENV-2 RNA was decreased to 0.46 ± 0.27-fold and 0.18 ± 0.06-fold after treatment with 62.5 and 125 μg/mL of *C. nutans*, respectively (Figure 2B). The levels of E antigen percentages were reduced dramatically by 48 h after treatment with *C. nutans* extract at 62.5 and 125 μg/mL by 70.19 ± 10.80 and 52.36 ± 13.62%, respectively (Figure 2C), in accompaniment to the IFA result which showed the reduction in infected cell numbers in *C. nutans* extract-treated cells (Figure 2D). These results confirmed the effect of *C. nutans* extract in inhibiting dengue virus in the early step of virus infection.

### 2.3. Treatment of C. nutans Extracts Inhibited the Virus Binding to Host Cells and Virus RNA Synthesis

**Figure 3:**
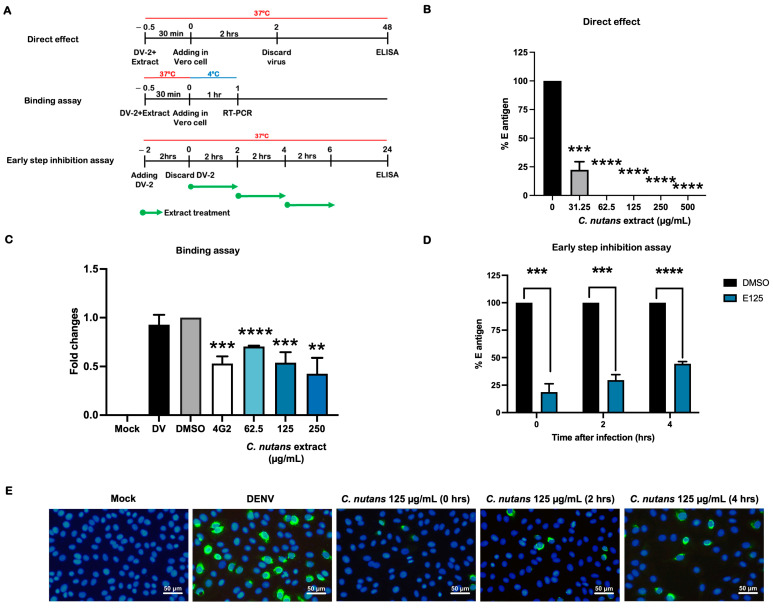
Effects of *C. nutans* extract on virus binding and early step of infection. (**A**) The scheme represents the protocol used to determine the direct effect, virus-receptor binding assay, and early-step inhibition assay (**B**). The direct effect of the extract on virus infectivity was determined by incubating DENV with varying concentrations of *C. nutans* extracts prior to the infection. The rate of infection was measured by cell-based ELISA (**C**). A virus–receptor binding assay was conducted to determine the extract’s effect on virus binding ability. The virus–extract complexes were added to the cells at 4 °C. The amounts of bound viruses were measured by using real-time PCR (**D**,**E**). An early-step inhibition assay was conducted to monitor the effect of *C. nutans* extracts on the early step of virus life cycle. The extract was added to the infected cells at 0, 2, and 4 h post-infection. The infection rate was determined by using cell-based ELISA and IFA. The results show the mean ± SEM values from three independent experiments (** *p* < 0.01, *** *p* < 0.001, **** *p* < 0.0001).

To assess the effect of the *C. nutans* extract on the steps of infection, the direct effect of the extract on DENV infection was determined (Figure 3A,B). We hypothesized that the extract might directly affect the virus, partially contributing to its ability to reduce DENV infection. The varying concentrations of *C. nutans* extract were incubated with DENV before infection. At the lowest concentration (31.25 μg/mL), the extract reduced the infection to 22.25 ± 12.38%, and higher doses completely inhibited the infection (Figure 2B). This indicated the extract’s ability to attenuate the virus. We further investigated whether the *C. nutans* extract affected the virus capability to bind to the host cells. The virus was incubated with the extract, and we allowed the complex to bind to the host cells at 4 °C. The amount of virus on the cell surface was determined by measuring their genomic RNA using real-time PCR. Incubating the virus with the extract significantly reduced the virus binding to host cells in a dose-dependent manner. Concentrations of 62.5, 125, and 250 μg/mL significantly decreased the virus binding to 0.70 ± 0.01-fold, 0.59 ± 0.18-fold, and 0.42 ± 0.28-fold, respectively (Figure 3C). The result suggested that the treatment affected the binding of the virus to the host.

Furthermore, our previous data showed that the extract also affected the virus during the post-infection step. We thus investigated the effect of the extract on protein translation and RNA synthesis, which occur in the early time point after virus entry. The extract was added at 0, 2, or 4 h after the infection. The result showed that the highest efficiency was observed when the cells were exposed to the extract immediately after the virus entered the cells, with reduced effectiveness over time (Figure 3D,E). Treatment after infection reduced the infection to 18.69 ± 13.20%, while treatment at 2 and 4 h post-infection inhibited infection to 29.60 ± 8.64% and 44.50 ± 3.56%, respectively (Figure 3D). This suggests that the *C. nutans* extract affected protein translation and RNA synthesis.

### 2.4. Reduction in Inflammatory-Related Gene Expression in DENV-2 Infected Cells by C. nutans Extracts

**Figure 4:**
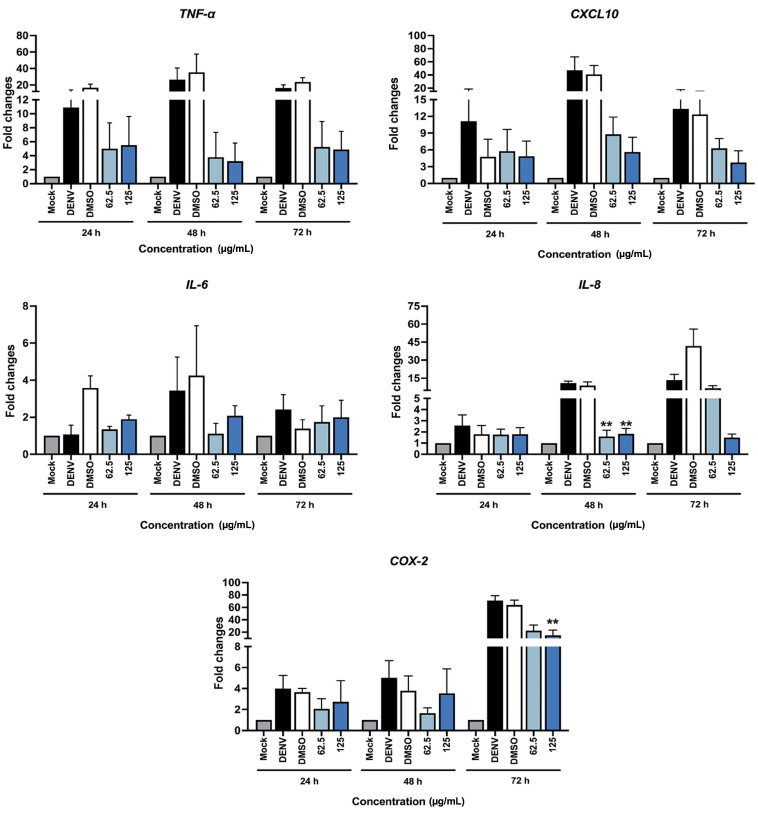
*C. nutans* extract reduces cytokine/chemokine production in DENV-infected Huh7 cells. Huh7 cells were infected with DENV-2 at an MOI of 0.1 and treated with the *C. nutans* extract at concentrations of 62.5 and 125 μg/mL for 24, 48, and 72 h. The transcription of *TNF-α*, *IL-6*, *IL-8*, *CXCL10*, and *COX-2* genes in the infected cells was determined by qRT-PCR. The expression levels were normalized with GAPDH and calculated relative to those of untreated DENV-infected cells. After 48 h of *C. nutans* extract treatment, a significant lowering of IL-8 gene expression was observed. In addition, after 72 h of *C. nutans* extract treatment, the expression of the *COX-2* gene was significantly lowered (** *p* < 0.01).

DENV infection is known to induce pro-inflammatory cytokines/chemokines and inflammatory enzymes, which are associated with the severity of dengue fever disease [21]. According to the previous report of anti-inflammatory activity of *C. nutans*, we expected that the *C. nutans* extract would lower cytokine/chemokine production in Huh7 cells after DENV-2 infection. The expression of anti-inflammatory-related genes was investigated by real-time PCR. The results demonstrated the upregulation of pro-inflammation genes including *TNF-α*, *CXCL10*, *IL-8*, *IL-6*, and *COX-2* after DENV infection. At 48 h of infection, the gene expression of *CXCL10*, *TNF-α*, *IL-8*, and *IL-6* was increased up to 47.14-fold, 26.43-fold, 10.87-fold, and 3.45-fold, respectively, while the *COX-2* gene was expressed 70.70-fold after 72 h of dengue viral infection (Figure 4). Interestingly, *C. nutans* extract could suppress the inflammation by lowering the transcription of *CXCL10*, *TNF-α*, *IL-8*, and *IL-6* to 8.77-fold, 3.78-fold, 1.59-fold, and 1.11-fold, respectively, after treatment with the *C. nutans* extract at 62.5 μg/mL for 48 h. Moreover, after treatment with the extract at 125 μg/mL for 48 h, the transcriptions of *CXCL10*, *TNF-α*, *IL-8*, and *IL-6* were reduced to 5.63-fold, 3.21-fold, 1.82-fold, and 2.08-fold, respectively. A reduction in *COX-2* expression was observed at 72 h of infection by lowering the gene expression to 22.53-fold and 15.13-fold when treated with the extract at 62.5 and 125 μg/mL, respectively.

### 2.5. C. nutans Extract Suppressed the Inflammation of DENV-Infected Cells by Inhibiting the NF-κB Signaling Pathway

**Figure 5:**
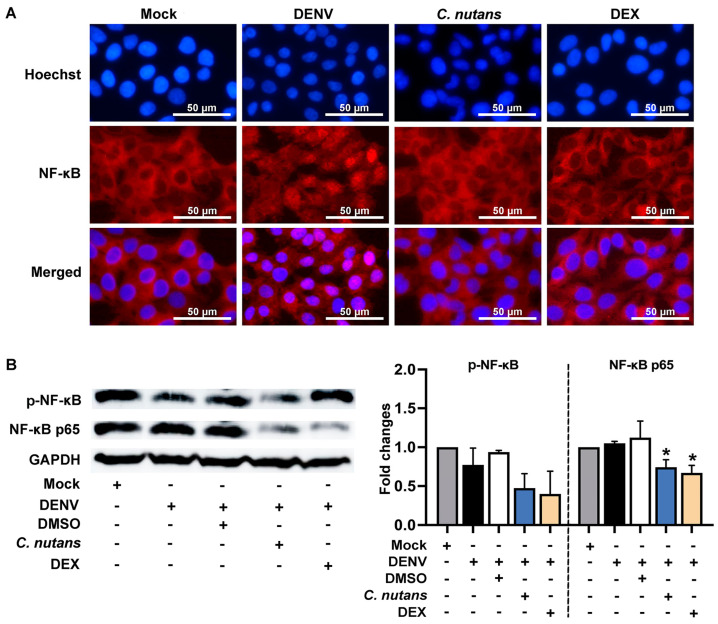
*C. nutans* extract suppressed NF-κB p65 signaling. Huh7 cells were infected with DENV-2 at an MOI of 0.1 and treated with 125 μg/mL of *C. nutans* extract, whereas 200 μM of DEX was used as a positive control. After 24 h of infection, the NF-κB p65 nuclear translocation was determined (red) and the nucleus was located with Hoechst staining (blue) (**A**). The protein expression of NF-κB p65 and its phosphorylated form was determined by immunoblotting (**B**). Protein band intensity was normalized to GAPDH by ImageJ software and calculated as a fold-change relative to the mock control (* *p* < 0.05).

To investigate the mechanism of *C. nutans* extract in lowering inflammation, we determined its effect on disturbing NF-κB signaling activation during DENV-2 infection. DENV infection activated the NF-κB cascade as evidenced by NF-κB nuclear translocation, which dramatically increased after the virus infection compared to the mock control (Figure 5A). As expected, NF-κB nuclear translocation was remarkably blocked by *C. nutans* extract treatment (Figure 5A). To confirm the inhibitory effect of *C. nutans* extract, we determined the NF-κB p65 protein levels and its phosphorylation after DENV infection compared to the conditions with or without *C. nutans* extract treatment (Figure 5B, Figure S2). In concordance with the nuclear translocation, the extract at the concentration of 125 μg/mL effectively decreased the protein level of NF-κB and its phosphorylated form by 0.74 ± 0.17-fold and 0.47 ± 0.26-fold, respectively (Figure 5B, Figure S2).

### 2.6. Gallic Acid Was a Bioactive Compound of C. nutans Extract

**Figure 6:**
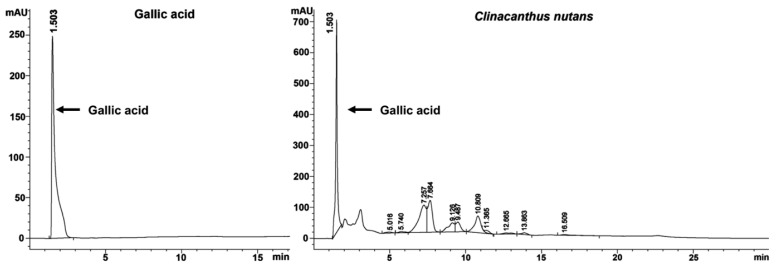
The gallic acid constituent in *C. nutans* extract. HPLC chromatograms of both the gallic acid standard and *C. nutans* extract were detected at 267 nm. A peak at the retention time of 1.503 min was observed in both the gallic acid standard and the *C. nutans* extract.

The bioactive compounds of *C. nutans* extract were reported in several studies. Interestingly, gallic acid found in the ethanolic extract of *C. nutans* has been reported for its antiviral activities against DENV [22]. We thus hypothesized that gallic acid is present in *C. nutans* extract and might be responsible for the anti-DENV activity of *C. nutans.* The total phenolic content of *C. nutans* extract was analyzed, which was found to be 27.44 ± 7.76 mg gallic acid/g extract. We further identified gallic acid as a representative bioactive compound in *C. nutans* extract by using HPLC. The results showed that gallic acid was a major compound that was identified by absorption spectra of relevant peaks and retention times compared with the gallic acid standard reference. The HPLC chromatograms showed a distinct peak of gallic acid at 1.503 min. Similarly, the chromatograms of the *C. nutans* extract also showed a dominant peak at 1.503 min (Figure 6).

### 2.7. Gallic Acid Inhibited DENV Infection

**Figure 7:**
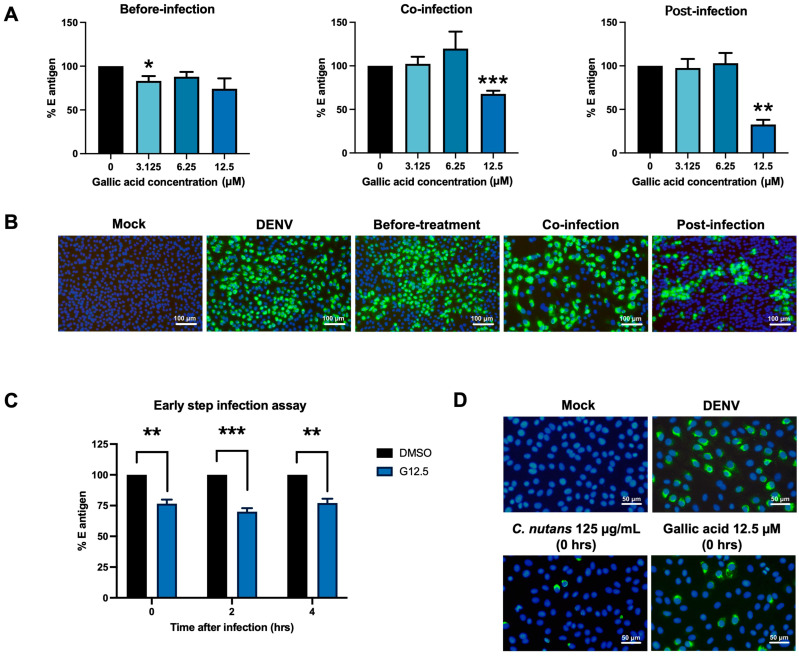
Gallic acid inhibited DENV infection. Gallic acid at concentrations of 3.125, 6.25, and 12.5 μM was applied to DENV-2 infected cells under different conditions: before-infection, co-infection, and post-infection conditions. The antiviral activity of gallic acid on reducing DENV infection was determined by cell-based ELISA (**A**) and IFA (**B**). An early-step infection assay was performed to investigate the effect of gallic acid after DENV entry by using cell-based ELISA (**C**) and IFA (**D**). The results show the mean ± SEM values from three independent experiments (* *p* < 0.05, ** *p* < 0.01, *** *p* < 0.001).

The antiviral activity of gallic acid against DENV infection has been reported in several studies. To confirm the activity of gallic acid and investigate the possible mechanism, we performed a time-of-addition assay in which gallic acid was applied to the cell in different conditions; before infection, co-infection, post-infection (Figure 7). In conjugation with *C. nutans* extract, gallic acid exhibited a stronger inhibitory activity on DENV infection during co-infection and post-infection conditions compared to the before-infection condition (Figure 7A). The greatest potential activity of gallic acid was found during the post-infection condition by reducing the relative E antigen to 32.71 ± 9.23% at the concentration of 12.5 μM, while the treatments during co-infection and before infection resulted in reductions of 67.76 ± 6.31% and 74.2 ± 20.58%, respectively. The inhibitory effect of gallic acid was confirmed by using IFA, which showed the highest reduction in infected cells during the post-infection condition, followed by the co-infection and before-infection treatments (Figure 7B). Additionally, we performed the early-step inhibition assay to further investigate the effect of gallic acid at various post-infection time points (0, 2, and 4 h post-infection). Although significant reduction in DENV infection was observed, there were no significant differences in the inhibitory activities among different time points (Figure 7C,D).

**Figure 8:**
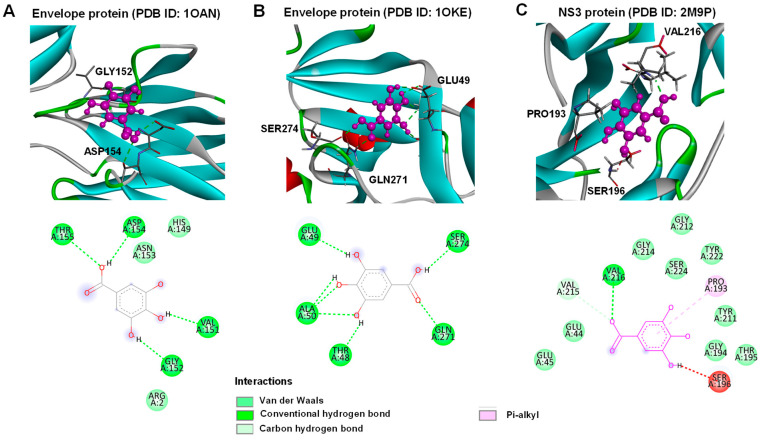
The interactions of dengue 2 virus envelope proteins and NS3 protease when docked with gallic acid. (**A**) Dengue 2 virus envelope protein [23]; (**B**) Dengue 2 virus envelope protein [24]; (**C**) Dengue NS3 protease [25] were docked with gallic acid using GOLD Protein–ligand docking software. Gallic acid is represented in magenta (**top**) and the detailed interacting residuals are presented in 2D diagram (**bottom**). All figures were visualized using Discovery Studio software.

Molecular docking was performed to further investigate gallic acid antiviral activity to target potential viral proteins, and DENV envelope proteins and NS3 protease were selected as they are crucial viral proteins involved in attachment and protein synthesis. Based on the analysis, gallic acid could bind to envelope proteins and NS3 protease as shown in Figure 8. The fitness scores for 1OAN, 1OKE, and 2M9P were 38.72, 41.98, and 41.71, respectively. The envelope protein 1OAN interacted with gallic acid on VAL151, GLY152, ASP154, and THR155 residuals with hydrogen bonds (Figure 8A). The other target site of the envelope protein, the hydrophobic pocket [23], interacted using the hydrogen bonds with THR48, GLU49, ALA50, GLN271, and SER274 residues. In the case of NS3 protease, the interaction was on PRO193 using Pi-alkyl and VAL216 using a conventional hydrogen bond (Figure 8C). These results suggest that gallic acid could interact with key viral proteins and could potentially be one of the bioactive compounds from *C. nutans* extracts. Certainly, this further supports the findings from the time-of-addition assay that demonstrated the antiviral activity of gallic acid, and the mechanism could be that gallic acid bound to the envelope proteins and viral protease.

## 3. Discussion

Reported cases of DENV infection are increasing continuously, and a large part of the world’s population is affected. Approaches to preventing the spread of DENV, as well as effective therapeutic treatments, are still needed to control the situation and reduce the risk of death. In the present study, the effects of *C. nutans* extract on decreasing viral replication and reducing the inflammation induced by DENV-2 infection in Huh7 cells were elucidated.

The efficiency of *C. nutans* extract in inhibiting DENV infection was determined during different steps of infection; before infection, co-infection, and post-infection (Figure 1A–C). The treatment with *C. nutans* extract at the co-infection step was the most effective in inhibiting DENV infection (Figure 1B), suggesting that the *C. nutans* extract might act during the early step of infection, i.e., viral binding and internalization. Moreover, considering the treatment before viral infection where the extract was applied to the cell prior to infection, the *C. nutans* extract had less effectiveness to inhibit the viral infection, which suggested the extract could directly impact on the virus rather than the cell receptor. Allowing DENV to bind to the host cells and then treating those cells with *C. nutans* extract after the viral infection for 2 h caused a significant reduction in viral RNA assessed at 24 h after infection (Figure 2B). This reflected that the extract did not only inhibit the viral binding step but also the steps after internalization.

We further conducted experiments to investigate whether the extract could directly affect DENV by incubating the virus with extracts shortly before the infection. The results demonstrated that the virus–extract incubation attenuated the infectivity of DENV (Figure 3B). This finding supports our previous hypothesis that the extract did not block the host cell receptor but instead lowered the virus’s ability to bind to the cells. To further confirm this phenomenon, we conducted a virus–receptor binding assay to assess the virus’s capability to bind to the host cells after treatment with the extract (Figure 3C). In alignment with our previous results, treatment with the *C. nutans* extract led to a significant reduction in virus binding, which would impact the ability of DENV to enter host cells. The entry step of the virus is a promising target of antiviral inhibitors. The binding of the virus to the host cell receptor promotes receptor-mediated endocytosis, allowing the virus to enter the host cells. According to the broad-ranged tropism and host cells of DENV, a variety of host receptors have been reported at present, i.e., glycosaminoglycans (GAGs), mannose receptors, DC-SIGN, heat shock protein 90/heat shock protein 70, and TIM/TAM [26,27], etc. Virus entry through the clathrin-mediated endocytosis pathway has been reported as the main route of DENV to hepatic cells [28]. Apart from the classical entry mechanism, the protein claudin-1 has been shown to interact with the DENV prM/M protein and is necessary for the viral entry step [29]. Blocking virus entry through interfering with the virus and host cell receptor binding is a well-known strategy for antiviral drugs or inhibitors. Previously, our group reported peptide inhibitors [30,31,32] and single chain variable fragment (ScFv) antibodies [33] to inhibit the interaction of the DENV envelope protein and host cell receptors, leading to a significant reduction in the DENV infection rate. Our result emphasized the effect of *C. nutans* on interfering with the virus–host binding. Previously, the effect of isolated bioactive compounds from *C. nutans* in disturbing the viral entry steps has been reported in other virus types. The purified monogalactosyl diglyceride (MGDG) and digalactosyl diglyceride (DGDG), extracted from *C. nutans* leaves, had the potential to inhibit HSV plaque formation [34]. Furthermore, two bioactive compounds called 136C and 136D from *C. nutans* leaves were shown to reduce the binding between protein receptors and human papillomavirus, as well as internalization [35]. DENV infection is a dynamic process. The virus enters the human body through a mosquito bite, replicates, and releases new virions into the bloodstream. DENV can circulate and infect various cell types depending on tissue tropism, initiating its life cycle by entering new host cells and releasing new virions. Although the *C. nutans* extract was most effective at the stage before viral entry, it is crucial to inactivate the virus and block its entry, thereby reducing the overall viral titer. This reduction can lower the risk of disease progression and severity. Moreover, decreasing the active virus in the bloodstream helps to interrupt the transmission cycle of dengue virus, which spreads among humans through mosquito bites, underscoring the approach’s impact on controlling disease transmission.

On the other hand, the effect of the *C. nutans* extract was also observed in the post-infection conditions; thus, we cannot exclude the possibility that the extract partially affected the other later steps such as RNA replication, protein translation, virus assembly, and virus maturation [36]. We conducted an early-step inhibition assay to analyze the potential impact of the *C. nutans* extract on viral infection. The extract was applied to cells at 0, 2, or 4 h post-infection, coinciding with the time frame of viral protein translation and RNA synthesis. Our results demonstrated a significant inhibitory effect of the extract on virus infection, which decreased over time (Figure 3D). The most effective inhibition occurred when cells were treated immediately after infection, while treatment at 4 h post-infection showed reduced effectiveness. Considering the correlation between extract efficiency and treatment timing, we hypothesize that the extract may inhibit early events, such as protein translation or RNA synthesis, rather than late-stage processes.

The uncontrolled response of the host cells after viral infection leads to massive cytokine/chemokine production, resulting in a cytokine storm, which is a well-known hallmark of severe DENV infection [37]. Clinical studies have shown a relationship between disease severity and increased pro-inflammatory cytokine/chemokines in the patient’s blood circulation [38]. Imad and colleagues studied the cytokine profile of 96 hospitalized adult patients admitted to the Hospital for Tropical Diseases, Bangkok, Thailand, during 2015–2016, which showed the increased expression of protein interleukins (IL-4, -6, -8, -10), TNFα, and IFNγ during infection [38]. Apart from the cytokine production, signs of severe hepatitis have been observed in these patients with elevated liver enzymes with an AST/ALT ratio over 400 U/L [38]. Interestingly, those with an AST/ALT ratio over 1000 U/L have a strong association with an increase in IL-8 during the acute phase and defervescence [38]. Liver injury including hepatomegaly, jaundice, increased liver aminotransferase enzymes, and acute liver failure has been reported as the warning sign for severe DENV and found in up to 60%–90% of dengue hemorrhagic fever (DHF) patients [39]. In accompaniment to the reported data, we demonstrated the direct effect of DENV infection in causing increases in *TNF-α*, *IL-6*, *IL-8*, and *CXCL10* expression in Huh7 cells (Figure 3). Importantly, the treatment with the *C. nutans* extract could remarkably lower the expression of these pro-inflammatory cytokines, especially *IL-8* which was significantly reduced at the mRNA level (Figure 4) and in protein secretion (Table S1). Moreover, the *C. nutans* extract treatment could reduce the expression of *COX-2*, which is responsible for the inflammation pathway (Figure 4). We thus expected that the treatment with *C. nutans* extract would help to ameliorate liver injury after DENV infection.

*C. nutans* extract treatment was shown to suppress the signaling of the NF-κB pathway by lowering the protein expression of NF-κB and phosphorylated NF-κB and inhibiting its activation by blocking the nuclear localization (Figure 5). NF-κB signaling was reported to contribute to the inflammation in DENV-infected cells and those correlated with the DENV NS5 translocation to the nucleus [40]. Inhibition of DENV and NS5 nuclear translocation by alpha-mangostin showed to suppress NF-κB signaling resulting in downregulation of the NF-κB responsive inflammatory genes including *RANTES*, *IP-10*, *TNF-α*, and *IL-6* production [40]. It is possible that the *C. nutans* extract treatment lowered the viral infection and corresponded to viral proteins which decreased the magnitude of inflammation activation in DENV infection maybe by reducing DENV NS5 protein level and its translocation. On the other hand, *C. nutans* extract could directly block the inflammation pathway since many phytochemical compounds from *C. nutans* ethanolic extracts have been reported for their anti-inflammation activities such as schaftoside, isoorientin, orientin, isovitexin, and vitexin, etc. [41]. The anti-inflammation activity of schaftoside was evidenced by its ability to reduce the gene expression of *IL-1β* and *IL-6* and *TNF-α* at the mRNA and protein levels in microglia [42,43]. The vitexin compound was reported to suppress the expression of IL-1β, IL-6, IL-33, TNF-α, and IL-10 in peripheral tissues and reduce MCP-1, IL-6, IL-8, TNF-α, CXCL1, and CX3CL1 in endothelial cells through the NF-κB and Nrf2 pathways [44,45,46,47]. Moreover, isovitexin and orientin were shown to reduce IL-1β, IL-6, IL-18, and TNF-α, as well as the corresponding elements in the inflammation pathway in RAW 264.7 macrophages upon LPS stimulation, i.e., prostaglandin E2, nitric oxide, and COX-2 via the NF-κB pathway [48,49]. Taken together, the anti-inflammation mechanism of *C. nutans* could either be a direct effect caused by the suppression of the inflammatory pathway or caused by its ability to protect the cells from viral infection.

To date, the characterization of *C. nutans*, particularly its leaves, has been well-documented by several groups, including our own. Based on the characterization by Lin et al., seven major bioactive components in *C. nutans* ethanolic extracts were identified, including schaftoside, orientin, vitexin, isoorientin, isovitexin, 6,8-apigenin-C-α-L-pyranarabinoside, and gallic acid (Table S2). Among them, only isoorientin, isovitexin, and gallic acid have been reported for anti-DENV activity in different cell types and anti-inflammatory activity in different disease models, not specifically in DENV infection. Indeed, phenolic compounds of medicinal plants have been reported for their capacity to inhibit DENV infection and exert anti-inflammatory effects [22]. Previously, we reported gallic acid from the Triphala Ayurvedic formulation comprising of *Phyllanthus emblica*, *Terminalia chebula*, and *Terminalia bellirica* to inhibit DENV infection [22]. Interestingly, Khoo et al. showed gallic acid was the bioactive ingredient in a *C. nutans* ethanolic extract [50], highlighting the possibility of gallic acid being found in our *C. nutans* extract and exerting anti-DENV function. As expected, the HPLC profile showed gallic acid as the major compound in *C. nutans* extract with a similar retention time to the pure gallic acid reference (Figure 6). Our previous study showed gallic acid had the capacity to bind with DENV NS5 proteins (MTase and RNA-dependent RNA polymerase/RdRp), which later resulted in inhibition of the RNA synthesis of DENV [22]. In this study, the molecular docking of gallic acid with crucial viral proteins, the envelope proteins and NS3 protease, confirmed that these proteins may be potential inhibitory targets of gallic acid (Figure 8). In fact, gallic acid is not the only substance contributing to anti-dengue activity. When compared with the crude extract, gallic acid showed a higher toxicity to cells and lower effectiveness in reducing dengue infection, suggesting that the use of gallic acid alone as a drug should be concerning. This result supports the use of *C. nutans* extract over a single compound treatment. Notably, although gallic acid is a component of many extracts, the proportion of gallic acid in those herbs and its natural combination with other bioactive substances are crucial. These combinations play significant roles in the effectiveness of *C. nutans* extract due to their synergistic effects. This suggested that the use of traditional crude extracts might offer greater benefits compared to the isolated bioactive compounds, which can broadly impact the treatment of public health problems like dengue fever in low- and middle-income countries. However, to evaluate the potential of *C. nutans* extract, an in vivo study in an animal model is needed to demonstrate its effectiveness in controlling viral titer and preventing liver injury, which is a critical symptom in severe DENV cases.

## 4. Materials and Methods

### 4.1. Plant and Extraction

Leaves of *C. nutans* were collected from Chiang Mai, Thailand, and were classified by Dr. Narin Printarakul, Faculty of Science, Chiang Mai University (deposited voucher: Specimen No. CN001-003). After drying at 60 °C for 24 h in a hot air oven, they were extracted using 70% ethanol (1:20 g/v) by ground, macerated, and shaken at 160 rpm for 12 h at room temperature. Then, the solvent was evaporated using a rotary evaporator. After drying in a water bath, the dried extract was kept at −20 °C until use and dissolved in DMSO before analysis.

### 4.2. Cell Cultures and DENV Propagation

Vero and Huh7 cells were purchased from the American Type Culture Collection (ATCC) (Manassas, VA, USA). DMEM-F12 media and MEM media supplemented with 2 mM L-glutamine, and 10% (*v*/*v*) FBS (Gibco, Thermo Fisher Scientific, Waltham, MA, USA) were used to grow Huh7 and Vero cells, respectively. All cell types were incubated in 5% CO_2_ at 37 °C.

The DENV serotype 2 (DENV-2) strain 16681 was kindly gifted from Prof. Pa-thai Yenchitsomanus, Division of Molecular Medicine, Faculty of Medicine Siriraj Hospital, Mahidol University, Thailand. The propagation of DENV was conducted in the C6/36 cell line using Leibowitz-15 culture medium supplemented with 1% FBS and 10% tryptose phosphate broth (TPB) (Gibco, Thermo Fisher Scientific, Waltham, MA, USA) for 5 days. After that, the amount of DENV titer was investigated by using a focus-forming units (FFU) assay.

### 4.3. Cell Viability Assay

The toxicity of the *C. nutans* extract was evaluated using a cell viability assay which was performed using PrestoBlue™ Cell Viability Reagent (Invitrogen, Carlsbad, CA, USA). Briefly, Huh7 cells at 1.5 × 10^4^ cells per well were cultivated in 96-well plates overnight. After that, *C. nutans* extract (62.5–1,000 μg/mL) was added and further incubated for 48 h. DMSO was used as a negative vehicle control. Then, PrestoBlue™ reagent was added to determine cell viability by measuring the absorbance at 560 nm and 590 nm using a microplate reader. The cell viability was calculated relative to that of the non-treatment control using the following equation.

(1) $$\%\;Cell\;viability=\left(\frac{Absorbance\;of\;tested\;condition}{Absorbance\;of\;non\text{-}treatment\;control}\right)\times 100$$

### 4.4. DENV Infection and Time-of-Addition Assay

To determine the effects of *C. nutans* extract on the various steps of DENV infection in Huh7-infected cells, we performed a time-of-addition assay. The treatment of *C. nutans* was divided into three conditions including (1) before-infection, (2) co-infection, and (3) post-infection steps. Briefly, Huh7 cells (1.5 × 10^4^ cells/well) were plated in a 96-well plate overnight before the experiment. DENV-2 at an MOI of 0.01 was used to infect the cells with or without *C. nutans* extract (a concentration ranging from 31.25 to 250 μg/mL). In the before-infection step, *C. nutans* extract was added to the cells for 2 h before infecting with DENV-2 for another 2 h. In the co-infection step, DENV-2 and *C. nutans* extract were added at the same time to Huh7 cells for 2 h at 37 °C. In the post-infection step, DENV-2 was added and incubated for 2 h before treating with *C. nutans* extract. After the infection and treatment, the excess unbound virus was discarded, and the cells were washed with 1X PBS. After 48 h of infection, the amount of virus in supernatants was determined by FFU assay and the infected cells were detected by a cell-based enzyme-linked immunosorbent assay and immunofluorescence assay (cell-based ELISA).

### 4.5. Focus Forming Unit (FFU) Assay

The amount of virus was titrated and stained using FFU assay. Briefly, Vero cells (1.5 × 10^4^ cells/well) were plated in a 96-well plate overnight before the experiment. The virus in the culture supernatants was diluted using a 10-fold serial dilution and then added to the cells. The plate was incubated at 37 °C in a 5% CO_2_ incubator for 2 h. After that, infected cells were covered with 1.5% carboxymethylcellulose in 2% FBS MEM media. After 72 h of infection, cells were fixed and permeabilized with 3.6% formaldehyde in 1X PBS and with 0.2% Triton X-100 in 1X PBS, respectively, for 15 min at room temperature. After washing with PBS twice, the mouse monoclonal anti-DENV-E antibody (4G2) was added to the cells for at least 3 h. After washing with 1X PBS containing 0.1% Tween-20 (PBST) three times, rabbit anti-mouse IgG conjugated with HRP (Dako, Santa Clara, CA, USA) was added to the cells at a 1:2000 dilution. The plate was incubated at room temperature for 1 h in a dark place. The foci formation of infected cells was indicated after adding the 3,30-diaminobenzidine (DAB) substrate (Sigma-Aldrich Corporation, St. Louis, MO, USA). The number of foci were counted manually under a light microscope (Drawell BDS400; Shanghai Drawell Scientific Instrument Co., Ltd., Shanghai, China) and expressed as FFU/mL.

### 4.6. Cell-Based ELISA

The cell-based ELISA assay was performed to determine the effect of the extract on lowering the intracellular viral envelope (E) protein. The detection of DENV-2 infection was performed as described previously in the presence or absence of *C. nutans* extract. The viral infected cells were harvested after 48 h of incubation and fixed with 3.6% formaldehyde followed by permeabilization with 0.2% Triton X-100 for 15 min. The 4G2 monoclonal antibody was added to the cells for at least 3 h. After washing with 1X PBS containing 0.1% Tween-20 (PBST) three times, rabbit anti-mouse IgG conjugated with HRP (Dako, Santa Clara, CA, USA) was added to the cells at a 1:2000 dilution. The plate was incubated at room temperature for 30 min in a dark place. After washing with 0.1% PBST, the 3,3′,5,5′-tetramethylbenzidine substrate (Invitrogen, Carlsbad, CA, USA) was used as a detection reagent where 2 N sulfuric acid was used to stop the reaction. The absorbance was measured at 450 nm and the mock control was set as blank. The intracellular E antigen was calculated as a percentage following this equation:

(2) $$\%\;E\;antigen=\left(\frac{Absorbance\;of\;treatmemt\;condition}{Absorbance\;of\;non\text{-}treatment\;control}\right)\times 100$$

### 4.7. Immunofluorescence Study (IFA)

The number of infected cells was determined after *C. nutans* treatment using IFA. Huh7 cells (1.5 × 10^4^ cells/well) were plated in a 96-well plate overnight before the experiment. DENV-2 infection was performed as described previously with or without *C. nutans* extract. The infected cells were harvested after 48 h of incubation and fixed with 3.6% formaldehyde followed by permeabilization with 0.2% Triton X-100 for 15 min. The cells were stained with 4G2 monoclonal antibody for at least 3 h at 37 °C. After washing with 0.1% PBST three times, goat anti-mouse IgG conjugated with Alexa Fluor 488 (dilution 1:2000) (Invitrogen, Carlsbad, CA, USA) and Hoechst^®^ 33342 (dilution 1:10,000) (Invitrogen, Carlsbad, CA, USA) were added and incubated for 30 min in dark conditions at room temperature. After washing with 0.1% PBST, the stained cells were investigated under a fluorescence microscope (Nikon Instruments, Inc., Melville, NY, USA).

For the NF-κB (p65) nuclear translocation assay, DENV-2 infected cells with or without *C. nutans* treatment were harvested at 24 h. The cells were fixed and permeabilized as previously described before adding NF-κB (p65) (D14E12) XP Rabbit antibody (CST#8242, 1:100) (Cell Signaling Technology Inc, Boston, MA, USA). The plate was incubated for 1 h before adding donkey anti-rabbit IgG conjugated with Alexa Fluor 555 (dilution 1:2000) (Invitrogen, Carlsbad, CA, USA) and Hoechst^®^ 33342. The localization of NF-κB p65 in intracellular was detected under a fluorescence microscope.

### 4.8. Real-Time PCR

The real-time PCR was conducted to determine the effect of *C. nutans* extract on the virus internalization step. Briefly, Huh7 cells were pre-chilled and DENV-2 at an MOI of 0.05 was infected to the cells. The treatment was incubated at 4 °C for 30 min. Then, *C. nutans* extract (62.5 and 125 μg/mL) was added for 2 h at 37 °C. After 24 h of incubation, the infected cells were collected, and the amount of DENV-2 virus was quantified using qRT-PCR technique with specific dengue primers (Table S3). Furthermore, the number of DENV-infected cells and the reduction in intracellular E protein in infected cells were measured to confirm the inhibitory activity by IFA and cell-based ELISA as previously described.

To evaluate the effect of *C. nutans* extract on cytokine gene expression, Huh7 cells were infected with DENV-2 (MOI 0.1) and treated with *C. nutans* extract at 62.5 and 125 μg/mL for 24, 48, and 72 h. At the harvest times, the RNA was isolated using TRIzol reagent (Invitrogen, Carlsbad, CA, USA) and used as the template for reverse transcription using cDNA Tetro cDNA Synthesis Kit (Bioline Reagents Ltd., London, UK). Real-time PCR was performed using a real-time RT-PCR kit according to the manufacturer’s instructions (Luna^®^ Universal qPCR Master Mix, New England Biolabs, Ipswich, MA, USA) and specific primers (Table S3) to determine expressions of cytokine, chemokine, and inflammatory enzyme encoding genes were used. The expression was normalized with those of GAPDH and compared to the non-treatment control (set as 1.0). The data are represented in relative normalized expression values.

### 4.9. Immunoblotting Assay

The immunoblotting assay was performed to measure the effect of *C. nutans* extract on NF-κB expression and signaling inhibition. Huh7 cells (1 × 10^5^ cell/well) were cultured as a monolayer. Then, DENV-2 (MOI 0.1) was used to infect the cells as described above followed by 125 μg/mL of *C. nutans* treatment. Dexamethasone (DEX) (Sigma-Aldrich, MO, USA) at a concentration of 200 μM was used as a positive control of NF-κB signaling inhibition. The virus-infected cells were harvested at 24 h and lysed using the RIPA buffer. The NF-κB protein and its phosphorylated form were determined by specific primary antibodies: phospho-NF-κB (p65) (Ser536) (93H1) (CST#3033, 1:100), NF-κB (p65) (D14E12) XP (CST#8242, 1:100) (Cell Signaling Technology Inc, Boston, MA, USA). The protein expression was detected with a secondary goat anti-rabbit antibody conjugated with horseradish peroxidase (Abclonal, Woburn, MA, USA). The GAPDH protein was used as an internal control. The protein intensity was quantified by ImageJ software (version 1.52a) and compared with the non-treatment control.

### 4.10. Determination of Total Phenolic Content

The total phenolic content of the *C. nutans* extract was determined by using the standard Folin-Ciocalteu assay. After incubating 50% Folin–Ciocalteu’s reagent (Merck, Kenilworth, NJ, USA) with various concentrations of *C. nutans* extract for 1 h, the absorbance was measured at a wavelength of 725 nm. The total phenolic content was calculated against the gallic acid standard curve and expressed as mg of gallic acid equivalents per gram extract.

### 4.11. Determination of the Phytochemical Composition of C. nutans Extract by HPLC

HPLC was performed to study the phytochemical profile of *C. nutans* extract using an HPLC instrument (Agilent Technologies, Santa Clara, CA, USA) with a ZORBAX Eclipse XDB-C18 column (4.6 × 150 mm, 5 μm (Agilent Technologies)). The linear gradient system of the mobile phase (deionized water: methanol in a ratio of 70:30 (*v*/*v*)) at a flow rate of 1 mL/min for 30 min was used. The absorbance was collected at a UV photodiode array detector (267 nm) for the evaluation of the gallic acid peak.

### 4.12. Molecular Docking of Dengue 2 Virus Envelope Proteins and NS3 Protease with Gallic Acid

Crystal structures of two dengue 2 virus envelope proteins were obtained from the Protein Data Bank [23,24] along with a structure of dengue NS3 protease [25] (https://www.rcsb.org/ accessed on 5 November 2023). Molecular docking was performed using the GOLD Protein–ligand docking software (GOLD version 5.3.0). Upon molecular docking, hydrogens and waters were removed from the structures. The structure of gallic acid as a target ligand was obtained from MolView (https://molview.org/ accessed on 6 November 2023). The binding sites were referenced with those of ligands extracted from the reported structures. The fitness score for each protein–ligand interaction was obtained from ChemPLP. The resulting docking poses and detailed interactions of the amino acid residues and the ligand were visualized using Discovery Studio Visualizer.

### 4.13. Statistical Analysis

GraphPad Prism 9 Software (GraphPad Software, Inc., La Jolla, CA, USA, version 9.5.1) was used to analyze statistics. All data were tested at least in three independent experiments. A bar graph was used represent the mean ± standard error of mean (SEM). Significant differences in means were compared between the treated group and the non-treatment control using one-way ANOVA, followed by Tukey’s multiple comparisons tests. Statistical significance was considered when the *p*-value was less than 0.05.

## 5. Conclusions

This study demonstrated the dual antiviral and anti-inflammatory activity of *C. nutans* extract on DENV-infected Huh7 cells. *C. nutans* extract inhibited DENV at the early step of infection. The treatment also remarkably lowered the pro-inflammatory cytokine expression and inflammation induced by DENV through suppression of the NF-κB signaling cascade. The result from our study showed the benefit of *C. nutans* extract treatment during DENV infection.

## Data Availability

The data used to support the findings of this study are available from the corresponding author upon reasonable request.

## Supplementary Materials

The following supporting information can be downloaded at: https://www.mdpi.com/article/10.3390/antibiotics13080705/s1, Figure S1: The cytotoxicity of *C. nutans* extracts in Huh7 cell; Figure S2: Effect of *C. nutans* on lowering p-NF-κB p65 and NF-κB p65 protein expression; Table S1: The effect of *C. nutans* extract to inhibit inflammatory cytokine/chemokine of Huh7 infected cells; Table S2: Anti-dengue activity and anti-inflammation activity of bioactive compounds found in *C. nutans* extract; Table S3: Sequences of primers for the qRT-PCR analysis. References [22,51,52,53,54,55,56,57,58,59,60] are cited in the Supplementary Materials.
