# Supplementary material for: Clinacanthus nutans (Burm. f.) Lindau Extract Inhibits Dengue Virus Infection and Inflammation in the Huh7 Hepatoma Cell Line
Source: Antibiotics (Basel). 2024 Jul 28;13(8):705. doi: 10.3390/antibiotics13080705 (PMC11350823; doi:10.3390/antibiotics13080705)
Supplement: Supplementary file 1 [file antibiotics-13-00705-s001.zip › antibiotics-3051555-supplementary.pdf]

antibiotics

MDPI

Supplementary Materials

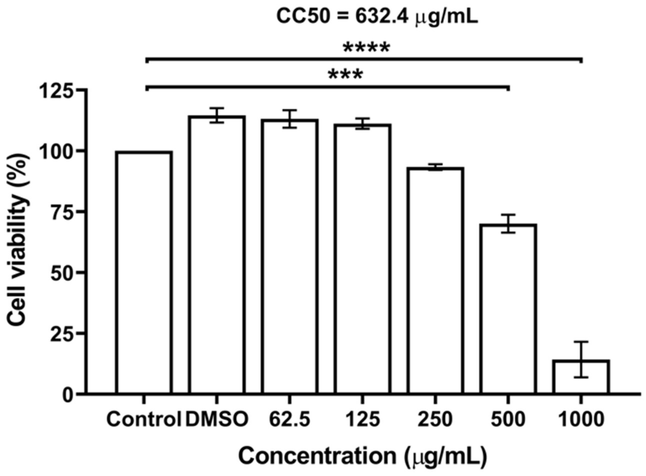

Figure S1. The cytotoxicity of *C. nutans* extracts in Huh7 cell. The concentration of *C. nutans* extract ranges from 62.5 - 1000 μg/mL was evaluated cell cytotoxicity in Huh7 cell using PrestoBlue™ Cell Viability Reagent after 48 h treatment. The cytotoxicity of *C. nutans* extract showed toxicity in a dose-dependent manner with the half-maximal cytotoxic concentration (CC50) being 632.4 μg/mL. Asterisks indicate a statistically significant difference from that of diluent control ***p < 0.001 and ****p < 0.0001).

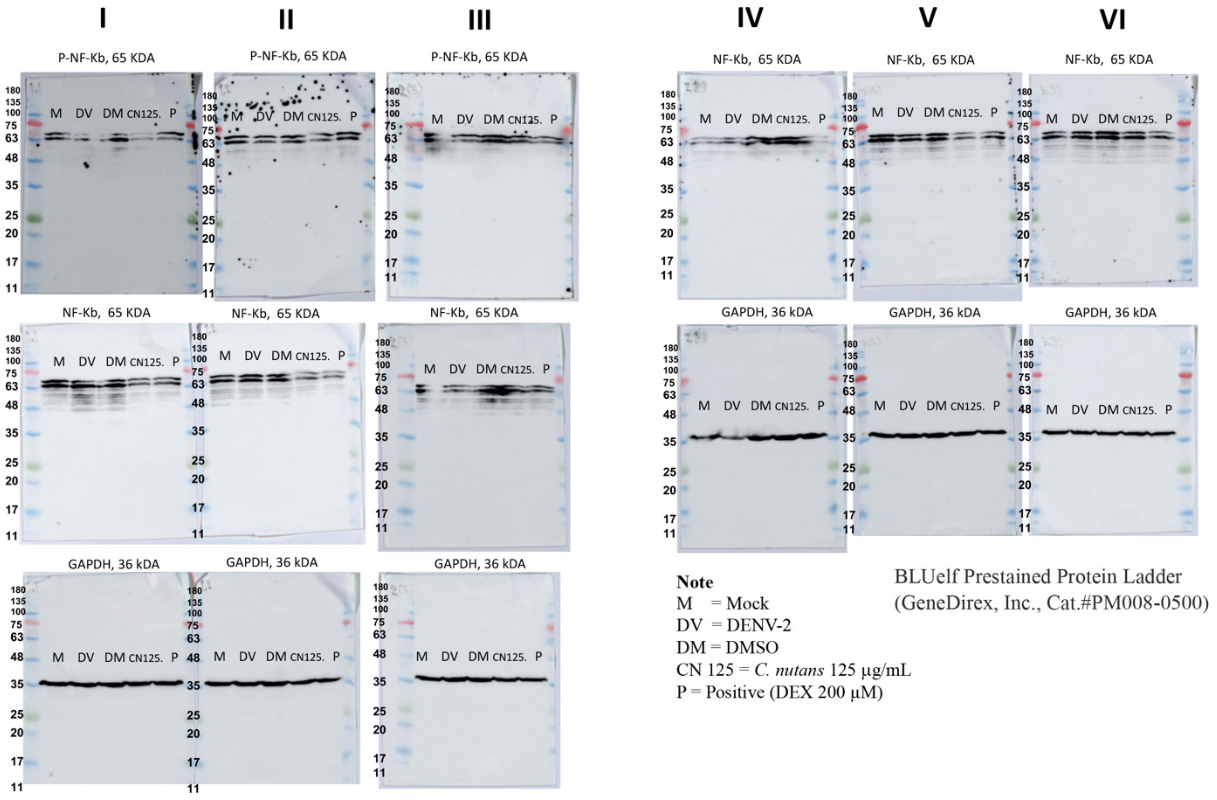

Figure S2. Effect of *C. nutans* on lowering p-NF-κB p65 and NF-κB p65 protein expression. The result of p-NF-κB p65 and NF-κB p65 proteins from at least three independent replications (I, II, II). Additional experiments for NF-κB p65 protein expression from other three independent replications (IV, V, VI).

Table S1. The effect of *C. nutans* extract to inhibit inflammatory cytokine/chemokine of Huh7 infected cells. The data were expressed as mean ± SD.

Treatments	IL-8 (pg/mL)
Mock	230.63 ± 107.44
DENV	220.00 ± 91.12
DMSO	175.55 ± 82.40
C. nutans 62.5 µg/mL	150.48 ± 84.92
C. nutans 125 µg/mL	107.14 ± 87.83

ND = Not be detected.

Table S2. Anti-dengue activity and anti-inflammation activity of bioactive compounds found in C. nutans extract.

Compounds [51]	Class	Anti-dengue activity	Anti-inflammation activity
Schaftoside	C-glycosidic flavone	[No], [52]	[Yes], LPS-induce B cell, [52]
Orientin	C-glycosidic flavone	[No], [53]	[Yes], LPS-induced HUVECs and mice, [53]
Vitexin	C-glycosidic flavone	[No], [54]	[Yes], human neutrophils, [55]
Isoorientin	C-glycosidic flavone	[Yes], Kidney cell and human lung fibroblast cell, [56]	[Yes], RAW 264.7 cell line, [57]
Isovitexin	C-glycosidic flavone	[Yes], Kidney cell and human lung fibroblast cell, [56]	[Yes], RAW 264.7 cells, [58]
6,8-apigenin-C-α-L-pyranarabinoside	C-glycosylflavone	[No], [59]	[No], [59]
Gallic acid	Steroid	[Yes], Vero cells and Hepatoma cells, [22]	[Yes], LPS-induced RAW264.7 cells, [60]

Table S3. Sequences of primers for the qRT-PCR analysis.

Primer	Sequence (5′-3′)
D2_ Forward	ATCCAGATGTCATCAGGAAAC
D2_ Reverse	CCGGCTCTACTCCTATGATG
CXCL10_ Forward	GAATCGAAGGCCATCAAGAA
CXCL10_ Reverse	AAGCAGGGTCAGAACATCCA
TNF-α_ Forward	TGCTTGTTCCTCAGCCTCTT
TNF-α_ Reverse	ATGGGCTACAGGCTTGTCACT
IL-6_ Forward	GTACATCCTCGACGGCATC
IL-6_ Reverse	AGCCACTGGTTCTGTGCCT
IL-8_ Forward	TCCTGATTTCTGCAGCTCTGT
IL-8_ Reverse	CCAGACAGAGCTCTCTTCCA
COX-2_ Forward	GCAGTTGTTCCAGACAAGCA
COX-2_ Reverse	GAAAGGTGTCAGGCAGAAGG
